# Robotische Hernienchirurgie I: Robotische Leistenhernienversorgung (r‑TAPP). Videobeitrag und Ergebnisse einer Kohortenstudie an 302 operierten Hernien

**DOI:** 10.1007/s00104-021-01425-6

**Published:** 2021-06-01

**Authors:** Michaela Ramser, Johannes Baur, Nicola Keller, Jan F. Kukleta, Jörg Dörfer, Armin Wiegering, Lukas Eisner, Ulrich A. Dietz

**Affiliations:** 1 Klinik für Viszeral‑, Gefäss- und Thoraxchirurgie, Kantonsspital Olten, Baslerstr. 150, 4600 Olten, Schweiz; 2 Klinik für Allgemein‑, Viszeral- und Gefässchirurgie, Kantonsspital Baden, Im Engel 1, 5404 Baden, Schweiz; 3 Hernienzentrum Zürich, Grossmünsterplatz 9, 8001 Zürich, Schweiz; 4 Klinik und Poliklinik für Allgemein‑, Viszeral‑, Transplantations‑, Gefäß- und Kinderchirurgie, Universitätsklinikum Würzburg, Oberdürrbacher Str. 6, 97080 Würzburg, Deutschland

**Keywords:** Leistenhernie, Minimalinvasive Leistenhernienversorgung, Lernkurve, Fascia transversalis, Serom, Groin hernia, Endoscopic groin hernia repair, Learning curve, Transverse fascia, Seroma

## Abstract

Die Versorgung von Leistenhernien mit offenen und minimal-invasiven Verfahren hat in den vergangenen 30 Jahren einen vom Ergebnis her gesehen hohen Standard erreicht. Allerdings besteht noch Bedarf an einer weiteren Reduktion der postoperativen Serome, chronischen Schmerzen und des Rezidivs. In diesem Videobeitrag wird die endoskopische Anatomie der Leiste im Hinblick auf die robotische transabdominelle präperitoneale Patchplastik (r‑TAPP) dargestellt und die Operationsschritte der r‑TAPP als Video illustriert. Es werden die Ergebnisse einer Kohortenstudie an 302 konsekutiven Hernien, die mittels r‑TAPP operiert wurden, vorgestellt und hinsichtlich des Mehrwerts der robotischen Technik – auch in der Weiterbildung – diskutiert. Die r‑TAPP ist die natürliche Weiterentwicklung der konventionellen TAPP und hat das Potenzial, bei zunehmender Geräteverfügbarkeit und Reduktion der Materialkosten zu einem neuen Standard zu werden. Künftige Studien werden den vielseitigen Mehrwert der r‑TAPP auch mit neuen Parametern verfeinern müssen.

**Video online:**

Die Onlineversion dieses Beitrags (10.1007/s00104-021-01425-6) enthält ein Video.

## Abstract (en)

The treatment of inguinal hernias with open and minimally invasive procedures has reached a high standard in terms of outcome over the past 30 years. However, there is still need for further improvement, mainly in terms of reduction of postoperative seroma, chronic pain, and recurrence. This video article presents the endoscopic anatomy of the groin with regard to robotic transabdominal preperitoneal patch plasty (r‑TAPP) and illustrates the surgical steps of r‑TAPP with respective video sequences. The results of a cohort study of 302 consecutive hernias operated by r‑TAPP are presented and discussed in light of the added value of the robotic technique, including advantages for surgical training. r‑TAPP is the natural evolution of conventional TAPP and has the potential to become a new standard as equipment availability increases and material costs decrease. Future studies will also have to refine the multifaceted added value of r‑TAPP with new parameters.

## Hintergrund

Vor 30 Jahren (1991) wurde mit der laparoskopischen Leistenhernienversorgung nach TAPP (transabdominelle präperitoneale Patchplastik) begonnen. In ihren Anfängen musste sich die TAPP gegen sehr gute offene Verfahren durchsetzen, die Operation war komplex, es mussten teure laparoskopische Systeme erworben werden. Die Gegenargumente waren stark: In der Shouldice-Klinik (Kanada) wurden seit den 1950er-Jahren Leistenhernien mit hervorragenden Ergebnissen ohne Netz und besser als nach Bassini operiert; im Lichtenstein-Institut (USA) war 1985 die spannungsfreie Netzreparation perfektioniert worden. Shouldice und Lichtenstein wurden unter Lokalanästhesie durchgeführt, waren kostengünstig und auch daher sehr überzeugend (siehe auch den Videobeitrag in *Der Chirurg* von Dietz et al. 2016; [1]).

Wie konnte sich die laparoskopische transabdominelle Netzplastik (TAPP) gegen so starke Argumente etablieren? Wie so oft bei neuen Entwicklungen in der Chirurgie, verdanken wir die TAPP nicht robusten präklinischen Daten, sondern visionären Pionieren, die die potenziellen Vorteile minimal-invasiven endoskopischen Arbeitens erkannten [2]. Erst Jahre später wurden die Ergebnisse der „optimalen TAPP“ (Arbeitsgruppe von Prof. Reinhard Bittner, Stuttgart) im Vergleich zum „perfekten Lichtenstein“ (Arbeitsgruppe von Prof. Henrik Kehlet, Kopenhagen) publiziert: Bei vergleichbarer Rezidivrate zeigten sich signifikant weniger chronische Schmerzen nach TAPP als nach Lichtenstein (*p* = 0,018; Odds Ratio [OR] 0,45; Konfidenzintervall [CI] 0,23–0,87; [3]).

Das Rezidiv nach TAPP ist allerdings ein Problem, das weiterer Verbesserung bedarf, eine exemplarische Studie zeigt für endoskopische Reparationen eine Rezidivrate von ca. 3,5 % [4]. Die HerniaSurge-Leitlinie (2018) macht darauf aufmerksam, dass mangelhafte Qualität der Hernienreparation einer der behebbaren Risikofaktoren für das Auftreten von Rezidiven ist, weshalb Weiterbildung, Standardisierung und Lernkurve so wichtig sind [5]. Suboptimale Ergebnisse können – gemessen an der Häufigkeit dieser Operationen – von großer sozioökonomischer Relevanz sein, nicht zuletzt, weil Lebensqualität und Optimierung des Körpers einen wichtigen Stellenwert in der Bevölkerung gewonnen haben. Hieraus ergeben sich zwei Schlussfolgerungen:

- Im nie endenden Kreis von Validierung und Falsifizierung müssen nach 30 Jahren TAPP neue Technologien Raum bekommen, um die Ergebnisse weiter zu verbessern, und
- es liegt in der Natur des Fortschritts, dass auch kleine Verbesserungen der Ergebnisse heute eines größeren Aufwands bedürfen als Ergebnisverbesserungen in der Vergangenheit.

Die robotische TAPP (r‑TAPP) ist so die natürliche Weiterentwicklung der konventionellen TAPP. Durch die Stabilität und Vergrößerung des Bildes, das ergonomische Arbeiten in einem weiten intraabdominellen Raum und den intuitiven Arbeitsmöglichkeiten der Präzisionsinstrumente gewinnen ChirurgInnen die Natürlichkeit des Dialoges mit dem Gewebe (Präparation) und die Freiheit im Schaffen von Synthese (Nähen und Knüpfen) in einer bisher in den minimal-invasiven Verfahren nicht bekannten Freiheit wieder.

In diesem Beitrag werden die Arbeitsschritte der r‑TAPP dargestellt und mit Ergebnissen aus dem eigenen Kollektiv veranschaulicht. Einleitend wird die posteriore Anatomie der Leistenregion repetiert und im Kontext robotischen Operierens im begleitenden Videobeitrag diskutiert.

## Indikationen und Kontraindikationen

Die Indikationen zur r‑TAPP sind prinzipiell ähnlich wie die zur konventionellen TAPP [5]. Die endoskopischen Vorteile der Inspektion des Darmes bei Inkarzeration sind auch für die Robotik gültig. Neu ist bei der Robotik, dass morbide Adipositas und Alter einen geringeren Einfluss auf die Verfahrenswahl haben als bei der konventionellen TAPP. Die Robotik hat einige merkbare Vorteile, die weit über Ergonomie, Freiheitsgrade der Instrumente, Bildstabilität und Immersionsblick hinausgehen, z. B.:

- Da die Ports an den Roboterarmen fixiert sind, wirken diese wie bei der früheren Lift-Laparoskopie gegen das Gewicht der Bauchdecke und ermöglichen eine merkbar bessere Arbeitsübersicht bei adipösen Patienten.
- Bei Bedarf kann bei kardiopulmonal belasteten Patienten aus dem gleichen Grund mit einem niedrigeren intraperitonealen Druck von z. B. 6–8 mm Hg gearbeitet werden.
- Der Abstand zwischen den Ports und dem Zielorgan ist immer konstant (und richtet sich z. B. nicht nach dem Bauchnabel), dadurch sind die Arbeitsbedingungen auch bei unterschiedlichen Körperbiotypen konstant reproduzierbar.

Dies erleichtert auch Eingriffe nach vorangegangen abdominellen Operationen, bei Vorhandensein von Stomata, bei Rezidiven oder nach Prostataresektionen.

Die Operationsindikation ist bei präoperativer Schmerzanamnese oder erhöhtem Schmerzrisikoprofil herausfordernd, oft ist eine präoperative Schmerzbehandlung anzustreben, da präoperative Schmerzen mit chronischen Schmerzen korrelieren [3]. Dies ist besonders bei jungen Patienten und Sportlern von Bedeutung, in einzelnen Fällen ist der bildmorphologische Ausschluss anderer Schmerzursachen (z. B. Adduktorentendinitis, Symphysitis, Lendenwirbelsäulen[LWS]-Syndrom) zu erwägen.

## Patientenaufklärung

Grundsätzlich gilt die gleiche Aufklärungssystematik wie für die konventionelle TAPP. Es wird das minimal-invasive Vorgehen dargestellt, mit der Versorgung aller potenziellen Bruchpforten mit Netz sowie der Option zur Mitversorgung der Leistenregion der Gegenseite oder konkomitanter Spieghel-Hernien. Es wird im Allgemeinen über postoperative Komplikationen wie postlaparoskopische Schulterschmerzen, Harnverhalt, Nachblutung, Serombildung und das Auftreten chronischer Schmerzen hingewiesen [6]. Die Punktionsstelle der Veres-Nadel links subkostal (erfunden wurde die Nadel vom ungarischen Internisten János Veres, 1903–1979) und die Rasur des Abdomens und des rechten Oberschenkels (für die Neutralelektrode) werden thematisiert. Als zu erwartende Rezidivrate werden die verfügbaren Ergebnisse der konventionellen endoskopischen Reparationen genannt (ca. 3,5 % auf 5 Jahre). Die Implantation eines in der Magnetresonanztomographie (MRT) sichtbaren nichtresorbierbaren großporigen Netzes wird besprochen.

Patienten mit Risikoprofil für chronische Schmerzen (z. B. bekanntes chronisches Schmerzsyndrom, junge Frauen oder mediterrane Ethnie) bekommen ein Rezept für Pregabalin, mit Beginn am Vorabend der Operation und Fortführung für weitere 3 Tage, zusammen mit der üblichen Schmerzmedikation (Paracetamol und Ibuprofen). Die Patienten werden über Optimierungsmöglichkeiten der postoperativen Narbenbehandlung beraten. Wir besprechen mit den Patienten auch den Einsatz des DaVinci Xi und erklären, dass es kein eigentlicher Roboter ist, sondern ein Präzisionsinstrument, das ausschließlich von den ChirurgInnen geführt wird.

## Anästhesie und Lagerung

Vor der Operation, in der Tagesklinik, erfolgt ein letztes Gespräch mit dem Patienten, es wird die Aufklärung kontrolliert und die zu operierende Seite am wachen Patienten mit Filzstift auf der Haut markiert. Der Patient wird in Rückenlage auf eine Antirutschmatte (Pink-Pad, Xodus Medical, USA) auf den Operationstisch (Trumpf Medical, Saalfeld, Deutschland) gelegt, ein Arm wird für die Narkose ausgelagert (z. B. der ipsilaterale Patientenarm zur Position des Roboters), Gesicht und Beatmungstubus werden mit einem am Operationstisch montierten Metallrahmen geschützt. Bei Anwendung des DaVinci-Xi-Systems (Intuitive Surgical, CA, USA) ist die anzusteuernde Patientenseite nicht relevant. In den allermeisten Fällen ist die Kopftieflage mit 10°-Trendelenburg-Lagerung ausreichend, bei sehr adipösen Patienten oder bei inguinoskrotalen Hernien ist eine 15°-Trendelenburg-Lagerung hilfreich. Der Eingriff wird unter Vollnarkose durchgeführt; die Relaxation muss bis zum Ende des Eingriffes bzw. bis zum Abdocken des Robotersystems optimal sein, bei Bedarf wird die neuromuskuläre Blockade am Ende des Eingriffes antagonisiert. Die Anästhesieausleitung erfolgt nicht im Operationssaal, damit dieser ohne Zeitverlust neu für den nächsten geplanten Eingriff gereinigt und aufgerüstet werden kann.

## Übersicht der endoskopischen Leistenanatomie

**Figure 1:**
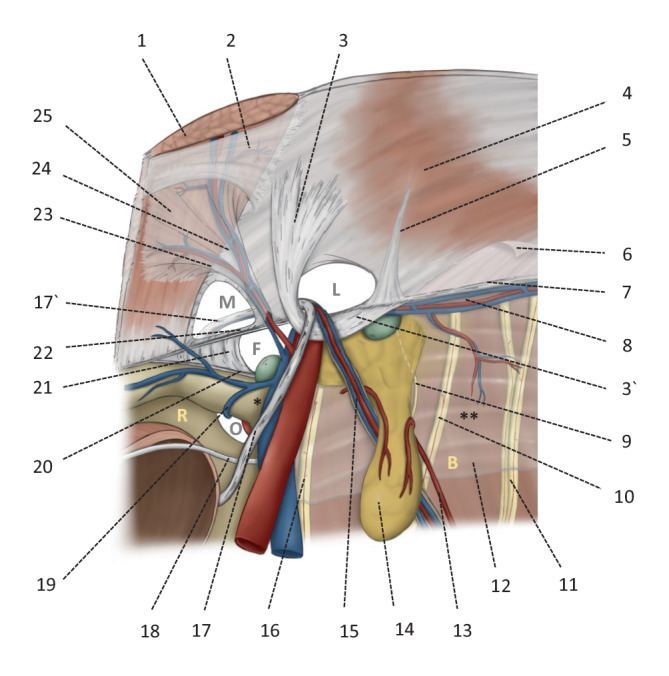

Die posteriore (endoskopische) Sicht auf die Leiste bietet nach dem französischen Chirurgen Henri Fruchaud (1894–1960) einen „Panorama-Blick“ auf den *myopektinealen Trichter,* welcher als anatomische Einheit unzertrennbar ist und die drei potenziellen Bruchlücken medial, lateral und femoral beinhaltet (Abb. 1). Bei jeder TAPP und r‑TAPP werden alle drei möglichen Bruchpforten systematisch dargestellt [7].

Das *Peritoneum* der Leistenregion haftet mit lockeren Bindegewebssträngen und teilweise durch eine Fettgewebsschicht lateralseitig dem M. transversus abdominis und medialseitig der hinteren Rektusscheide an. Der genaue Verlauf der Fascia endoabdominalis bzw. der Fascia intermedia sowie der Fascia recti propria muss in anatomischen Studien erst neu definiert werden, hier besteht noch keine abschließende Klarheit, weder von Seiten der Anatomie noch der Embryologie. Leitstruktur der endoskopischen Leistenanatomie ist die *A. epigastrica inferior* (Abb. 1/24). Medial der A. epigastrica inferior, lateral des M. rectus abdominis und kranial des Leistenbands ist das *Hesselbach-Dreieck* (benannt nach Franz Caspar Hesselbach, 1759–1816) lokalisiert; es wird von der Fascia transversalis ausgekleidet und ist der Ort medialer (direkter) Hernien (Abb. 1/M). Wahrscheinlich ist das, was von ChirurgInnen „Fascia transversalis“ genannt wird, die mediale Insertionsaponeurose des M. transversus abdominis, während die eigentliche „Fascia transversalis“ am ehesten Teil der größeren Fascia endoabdominalis ist [8]. Im aktuellen Text bezieht sich der Begriff Fascia transversalis auf die historische Bedeutung der Hinterwand des Leistenkanals, wie unter ChirurgInnen üblich. Die Auswölbung der Fascia transversalis bei medialen Hernien bildet den sog. *„outer sac“.* In diesem Bereich finden sich oft venöse Anastomosen zwischen der V. epigastrica inferior und den retropubischen Venen („rectusial veins“), dem sog. „venösen Circulus“ von Robert Bendavid (1940–2019); die Gefäßäste, die oberhalb des Hesselbach-Dreiecks von den epigastrischen Gefäßen nach medial verlaufen, sind die eigentliche *Corona mortis* (Abb. 1/23). Bei der notfallmäßigen Herniotomie in den Jahrhunderten vor der modernen Hernienreparation wurde der Leistenschnitt „von unten nach oben“ geführt, um den Leistenring zu erweitern und den inkarzerierten Darm zu befreien, wodurch bei medialen Hernien die innere Blutung an dieser Stelle oft zum Tode führte und die da verlaufenden Gefäße daher als Corona mortis bekannt wurden. Der Würzburger Prosektor Hesselbach (1759–1816) erfand hierzu eigens eine chirurgische Klemme mit Schraubverschluss, um Blutungen an der Corona mortis zu stillen. Auch wenn endoskopischen ChirurgInnen die retropubischen bzw. rektusialen Venen bedrohlich imponieren, entsprechen sie nicht der ursprünglichen Corona mortis.

Lateral der A. epigastrica inferior befindet sich der innere Leistenring (Abb. 1/L), begrenzt unterhalb vom Leistenband bzw. vom Tractus iliopubicus und oberhalb vom M. transversus und M. obliquus internus. Hier treten laterale (indirekte) Hernien aus. Nicht selten finden sich etwas lateral davon kaudale Spieghel-Hernien (benannt nach dem Brüsseler Anatomen Adrian van der Spieghel, 1578–1625). Bei lateralen Hernien verläuft der peritoneale Bruchsack mitsamt den *Testikulargefäßen* (Abb. 1/15) und dem *Ductus deferens* (Abb. 1/17) durch den Leistenkanal; bei der Frau verläuft das *Ligamentum teres uteri* durch den Leistenkanal. Der *Ramus genitalis des N. genitofemoralis* (Abb. 1/9) tritt in nur ca. 14 % der Fälle durch den Leistenkanal; meistens „durchbohrt“ er den Tractus iliopubicus (Abb. 1/22) oder tritt kranial dessen durch die Bauchdecke und findet seinen Weg zu den Kremasterfasern [9]. Im Leistenkanal am Bruchsack entlang verläuft der Ramus genitalis in der aus der offenen Hernienreparation bekannten „blue-line“, einer Testikularvene anliegend [10]. Das Peritoneum der lateralen Leistenhernie bildet den sog. *„inner sac“*. Bei 20–70 % der Patienten besteht ein *Lipom des Samenstrangs* (Abb. 1/14), was korrekterweise ein präperitonealer Fettprolaps und kein eigentliches Lipom ist; das Lipom präperitonealen Ursprungs ist kranial gestielt (Abb. 1/13) und ragt meist lateral des Bruchsacks und des Funiculus spermaticus in den Leistenkanal; es erhält seine Durchblutung von proximal des Leistenrings [11]. Bei ca. 8 % der Patienten besteht lediglich ein Lipom, ohne peritonealen Bruchsack, dieser Befund wird als EHS(European Hernia Society)-L1-Hernie klassifiziert [12]. Es gibt auch Samenstranglipome, die „perlenkettenartig“ und ohne erkennbaren Gefäßstil den Leistenkanal ausfüllen, diese entstehen wahrscheinlich aus dem Fettgewebe des Funiculus spermaticus.

Die *A. und V. iliaca externa* verlaufen mit dem *N. femoralis* (Abb. 1/16) sowie ggf. *Lymphknoten* (Abb. 1/grün) durch die Lacuna vasorum (Abb. 1/F) unter dem Tractus iliopubicus in den Oberschenkel. Medialseitig wird die Lacuna vasorum vom *Ligamentum lacunare* abgegrenzt (benannt nach dem spanischen Wundarzt Don Antonio de Gimbernat y Arbós, 1734–1816; Abb. 1/21), hier entsteht die Femoralhernie (Abb. 1/F). Das Ligamentum lacunare verbindet das Leistenband mit dem *Ligamentum pectineum* (benannt nach dem Londoner Chirurgen Sir Astley Paston Cooper, 1768–1841). Unterhalb des Leistenbandes und des inneren Leistenrings liegen iliakale Lymphknoten (Abb. 1/grün). Der Raum zwischen der Symphyse und der Harnblase ist als *Spatium Retzii* bekannt (benannt nach dem schwedischen Anatomen Anders Adolf Retzius, 1796–1860; Abb. 1/R); neuere anatomische Studien definieren den präperitonealen Retzius-Raum als Raum zwischen der Urogenitalfaszie (welche die Harnblase bedeckt) und der Fascia transversalis (welche Teil der Fascia endoabdominalis und nicht mit der Faszie des M. transversus zu verwechseln ist; [8]). Unterhalb des Ramus horizontalis des Os pubis verläuft der *Canalis obturatorius* (Abb. 1/O). Das Spatium Retzii mündet lateral der Iliakalgefäße in den präperitonealen *Bogros-Raum* (benannt nach dem französischen Anatomen Annet-Jean Bogros, 1786–1823; Abb. 1/B; [13]); hier verlaufen der *N. genitofemoralis* (Abb. 1/10; mit dem Ramus genitalis und dem Ramus femoralis) sowie der *N. cutaneus femorias lateralis* (Abb. 1/11), meistens unter der *Fascia iliaca* (Abb. 1/12); die Fascia iliaca und die Nerven müssen bei der Präparation unbeschädigt bleiben [9, 14, 15].

Bei der endoskopischen Präparation im Rahmen der rTAPP sind die Nn. ilioinguinalis und iliohypogastricus nicht sichtbar, sie verlaufen im Becken kranial der Spina iliaca anterior et superior und treten in den Raum zwischen Mm. obliqui internus und externus in die Leistenregion.

## Operationsschritte

**Figure 2:**
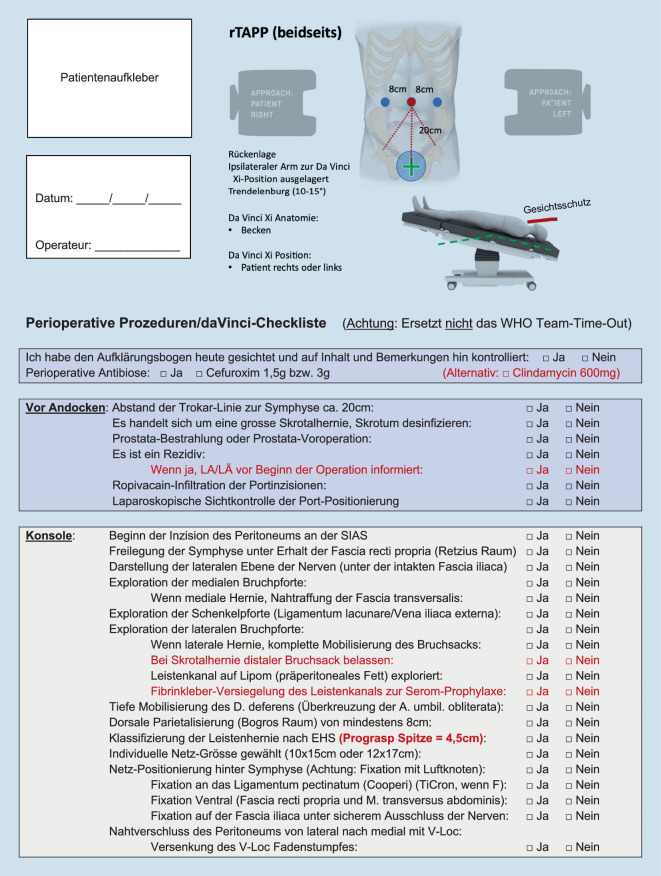

**Figure 3:**
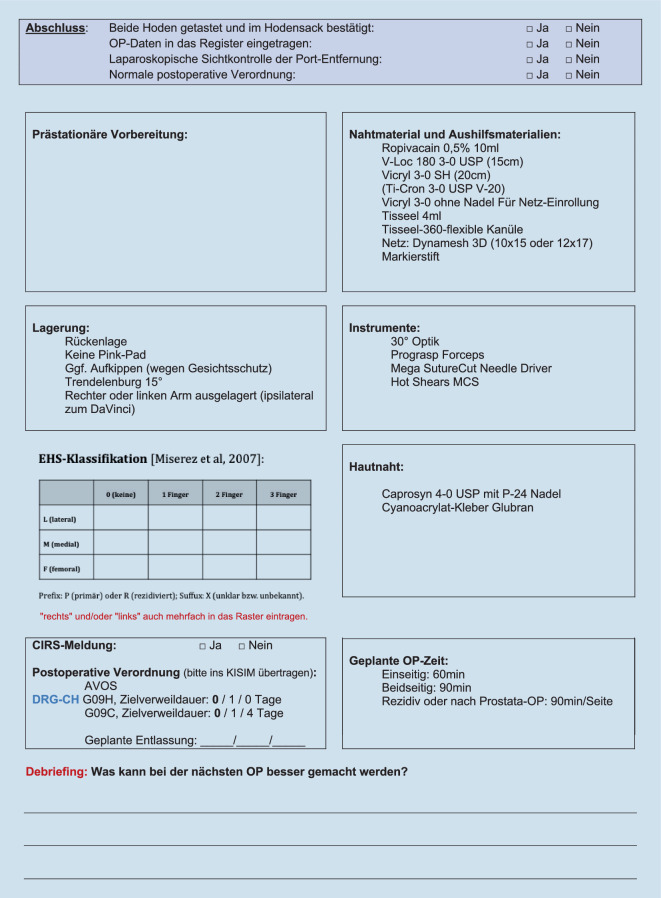

Begonnen wird mit dem WHO-Team-time-out, dem Repetieren der geplanten Arbeitsschritte sowie der Besprechung möglicher Abweichungen mittels standardisierter intraoperativer Checkliste (Abb. 2). Über eine Veres-Nadel wird das Pneumoperitoneum angelegt und die 3 Arbeitsports werden standardisiert positioniert (Abb. 3; Zusatzmaterial online Videosequenz 00:58–02:04 min). Es erfolgt ein Rundumblick mit diagnostischer Laparoskopie und das Andocken des Robotersystems und die Tischpositionierung in 10°-Kopftieflage (Trendelenburg). Der DaVinci Xi (Intuitive Surgical, CA, USA) und der Operationstisch (Trumpf Medical, Saalfeld, Deutschland) sind über Bluetooth gekoppelt, der Tisch kann bei Bedarf intraoperativ (z. B. bei sehr adipösen Patienten) bei angedocktem Roboter während der Operation nachpositioniert werden. Nach dem Andocken an die Ports werden die über dem Patienten schwebenden Roboterarme etwas deckenwärts versetzt, was den Radius des Abdomens erweitert und das Arbeiten mit geringerem Pneumoperitoneumdruck bei gleichem Raumvolumen ermöglicht (8–12 mm Hg). Da das Robotergedächtnis auf den sog. stationären Punkt der Ports achtet (schwarzer Ring am Portschaft), wird die Bauchdecke nicht geschädigt. Wir arbeiten mit dem DaVinci Xi an zwei Operationskonsolen. Standardmäßig werden die monopolare Schere (Hot Shears MCS), eine Fasszange (Prograsp Forceps) und der Nadelhalter mit integrierter Schere (Mega SutureCut Needle Driver) sowie eine 30°-Optik verwendet. Alternativ zum Prograsp Forceps kann eine bipolare Fasszange (Fenestrated Bipolar Forceps oder Maryland Bipolar Forceps) verwendet werden.

**Figure 4:**
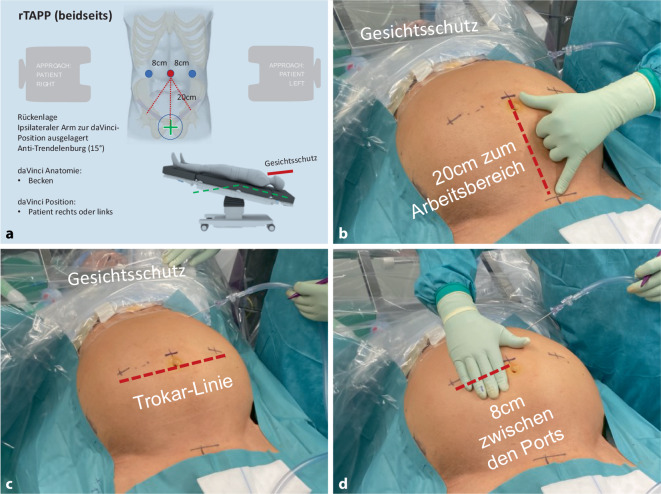

Die hier in 8 Abschnitte aufgeteilten Operationsschritte 1 bis 8 werden im Onlinevideo dieses Beitrages in der gleichen Systematik dargestellt und werden im Folgenden beschrieben (Abb. 4):

### 1. Schritt – Inzision des Peritoneums (Abb. 4a; Zusatzmaterial online Videosequenz 02:21–03:20 min).

Eröffnung des Peritoneums von lateral nach medial, beginnend in Höhe der Spina iliaca anterior et superior. Dieser Zugang erfolgt in weitem türflügelartigem Bogen, um ein ausreichend großes Netz positionieren zu können.

### 2. Schritt – Darstellung der Symphyse (Abb. 4b; Zusatzmaterial online Videosequenz 03:21–04:24 min).

Mediale Darstellung des M. rectus abdominis (unter Erhalt der Linea arcuata und der eigentlichen Fascia recti propria), der Symphyse und der Harnblase im Spatium Retzii (dieser Zugang unterscheidet sich von der TEP[totale extraperitoneale Plastik]-Technik, bei welcher der Einstieg durch die hintere Rektusscheide erfolgt). Der mediale Ansatz des Leistenbandes, das Ligamentum pectineum und das Ligamentum lacunare werden gesichtet. Die Symphyse wird ausreichend dargestellt, damit das Netz ca. 2 cm nach kontralateral überlappen kann.

### 3. Schritt – Darstellung der Nerven unter der Fascia iliaca (Abb. 4c; Zusatzmaterial online Videosequenz 04:25–05:10 min).

Laterale Darstellung der Ebene der Fascia iliaca, mit Sichtung der darunter verlaufenden Nerven (N. cutaneus femoris lateralis und dem N. genitofemoralis mit seinen Rami genitalis und femoralis). Hier ist der Bogros-Raum lokalisiert. In seltenen Fällen verlaufen die Nerven nicht unter der Faszie; durch den Beginn der Präparation von lateral können solche atypischen Nervenverläufe sicher erkannt werden.

### 4. Schritt – Präparation der Bruchpforten (Abb. 4d–g; Zusatzmaterial online Videosequenz 05:11–11:29 min).

Präparation des myopektinealen Trichters von lateral nach medial. Herauslösung des lateralen Bruchsacks („inner sac“) aus dem Leistenkanal, mit Abtrennung desselben von den mit Fett und lockerem Bindegewebe umhüllten Testikulargefäßen und dem Ductus deferens (Abb. 4d). Falls der Bruchsack sehr lang oder embryonal bis zum Skrotum eingewachsen ist, kann der distale Anteil belassen werden. Der Leistenkanal muss immer auf einen möglichen begleitenden Fettprolaps, das Samenstranglipom, kontrolliert werden (Abb. 4e). Bei großer lateraler Hernie wird der Leistenkanal zur Seromprophylaxe durch Aussprühen mit Fibrinkleber versiegelt (Tisseel 4 ml, Baxter, mit flexiblem Applikator; Abb. 4f; Zusatzmaterial online Videosequenz 08:41–09:17 min).

Medial der epigastrischen Gefäße wird die mediale Hernie präpariert; durch den Druck des Pneumoperitoneums wölbt sich die Fascia transversalis nach außen („outer sac“). Die Fascia transversalis wird mit einer fortlaufenden V‑Loc-Naht morphologisch rekonstruiert, die Hinterwand des Leistenkanals ist anschließend wieder geglättet (Abb. 4g; Zusatzmaterial online Videosequenz 09:27–10:53 min); dabei muss unbedingt auf den Verlauf der Testikulargefäße und den Ductus deferens geachtet werden, die hinter der Fascia transversalis im Leistenkanal zu finden sind, damit sie nicht „blind“ mitgefasst werden (Abb. 1/17’; Cave: Blutung und chronische Schmerzen). Medial der epigastrischen Gefäße und unterhalb des Leistenbandes wird entlang der V. iliaca externa die Lacuna vasorum präpariert und auf eine femorale Hernie hin untersucht (Zusatzmaterial online Videosequenz 10:56–11:24 min).

Nach Komplettierung der Präparation wird die Hernie nach EHS klassifiziert. An dieser Stelle wird auf die großen Iliakalgefäße geachtet, welche im Dreieck, das vom Ductus deferens und den Testikulargefäßen gebildet wird, verlaufen und als „triangle of doom“ bekannt sind (Abb. 1/*): Das unvorsichtige Präparieren an dieser Stelle kann zum (Blutungs‑)Verhängnis werden [16].

### 5. Schritt – Dorsale Parietalisierung (Zusatzmaterial online Videosequenz 11:30–12:58 min).

Der Ductus deferens wird bis tief ins kleine Becken gelöst. Nun erfolgt die Parietalisierung nach dorsokranial entlang der Mm. psoas und iliacus auf einer Strecke von mindestens 8 cm im Bereich des Bogros-Raums. Die Nerven bleiben dabei im Sichtfeld unter der Fascia iliaca und werden geschont.

### 6. Schritt – Einbringen des Netzes (Zusatzmaterial online Videosequenz 12:59–13:29 min)

Einbringen eines mindestens 10 × 15 cm großen Netzes (großporig, flach, MRT-sichtbar. Dynamesh, Aachen, Deutschland). Die Netzpositionierung beginnt retrosymphysär im Spatium Retzii, die Netzunterkante liegt lateral und dorsokranial im Bogros-Raum. Das Netz soll den Hauptbefund um ca. 5 cm überlappen. Je nach Befund muss ein größeres Netz verwendet werden (z. B. 12 × 17 cm). Bei beidseitiger Hernienversorgung überlappen die Netze medial um 2–3 cm.

### 7. Schritt – Netzfixation (Abb. 4h, i; Zusatzmaterial online Videosequenz 13:30–14:48 min).

Netzfixation mit lockeren resorbierbaren Nähten an 4 Punkten. Begonnen wird immer a) am Ligamentum pectineum (Abb. 4h), dann b) an der Fascia recti propria, c) am M. transversus abdominis und schließlich d) an der Fascia iliaca (Abb. 4i). Cave: Dieser letzte Fixationspunkt ist formal im „triangle of pain“ lokalisiert, der anatomische Bereich lateral der Testikulargefäße und unterhalb des Tractus iliopubicus (Abb. 1/**; [17]).

Die HerniaSurge-Leitlinie rät wegen des Risikos der Nervenverletzung von der Tackerfixation an dieser Stelle unbedingt ab. Unter Roboterarbeitsbedingungen kann allerdings die Fixation mit einem Luftknoten unter sicherer Aussparung der Nerven exakt an die Fascia iliaca gesetzt werden. Wir vertreten diese Fixation, da das Rezidiv endoskopischer Reparationen meistens genau hier zu finden ist. Bei Femoralhernien werden wegen der limitierten dorsalen Netzunterfütterung zwei nichtresorbierbare Nähte an das Ligamentum pectineum gesetzt.

### 8. Schritt – Naht des Peritoneums (Zusatzmaterial online Videosequenz 14:49–15:44 min).

Nahtverschluss des Peritoneums von lateral nach medial. Bei Verwendung monodirektional selbstsichernder Nahtmaterialien (z. B. V‑Loc/Medtronic, Deutschland oder Stratafix/Ethicon-Johnson&Johnson) muss der Fadenstumpf zum Ende der Naht unbedingt versenkt werden, da sich sonst am Fadenstumpf Darmverwachsungen bilden, die bereits nach 2 Tagen zu revisionspflichtigem Ileus führen können [18]. Die Nadeln und Fadenreste werden entfernt, es erfolgt die Zählkontrolle aller Operationsmaterialien. Die Faszienlücken im Bereich der drei 8‑mm-Ports müssen nicht verschlossen werden. Die Haut wird mit resorbierbarem Fadenmaterial intrakutan genäht und mit Cyanoacrylat-Kleber (alternativ Hydrokolloidverband zur optimalen Zugentlastung) versiegelt.

**Figure 5:**
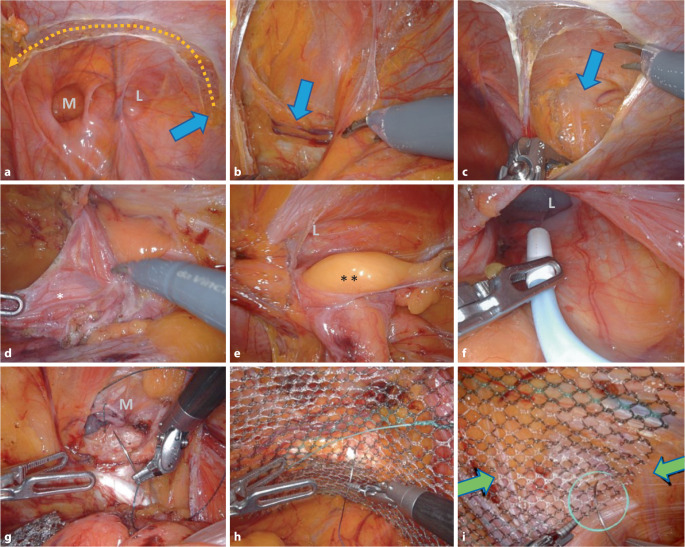

## Postoperative Behandlung

Die peri- und postoperative Analgesie ist sehr wichtig, um die Chronifizierung perioperativer Schmerzen zu vermeiden. Ambulante Patienten werden vom Operationssaal für 1–2 h in den Aufwachraum verlegt und anschließend über die Tagesklinik, wo sie noch einmal 2–3 h verbringen, entlassen. Vor der Entlassung stehen sie mithilfe der Pflege auf, gehen selbständig auf die Toilette und bekommen eine leichte Mahlzeit. Belastung und Sport sind nach Maßgabe der Beschwerden und so früh wie möglich zu empfehlen, eine spezifische Lastenrestriktion ist nicht nötig [19]. Das Nahtmaterial muss nicht entfernt werden. Die Narbenpflege mit UV-Blocker für 6 Monate und Massageroller wird von den Patienten geschätzt.

## Kasuistik und Studiendesign

Dieser Videobeitrag fasst die Erfahrungen an 302 konsekutiven r‑TAPPs zusammen, welche in einem Zeitraum von 18 Monaten operiert wurden. Es handelt sich um eine Kohortenstudie ohne Kontrollgruppe. Die Datenerfassung begann mit dem ersten Eingriff der Implementierungsphase des Robotikprogramms der Viszeralchirurgie am Kantonsspital Olten (KSO) und beinhaltet somit auch die Zeit der Lernkurve im Umgang mit dem Operationsroboter. Die Studie wurde von der zuständigen Ethikkommission der Nordwestschweiz bewilligt (Ref. Nr. 2019-02046).

Entscheidungen über Interventionen auf Ebene der Bruchpforten (Raffung der Fascia transversalis bzw. Fibrinkleberversiegelung des Leistenkanals) und Netzgröße wurden intraoperativ je nach Befund im Sinne des „tailored approach“ im Rahmen des üblichen Versorgungsauftrags gefällt. Die Patienten wurden im Voraus darüber aufgeklärt, dass je nach intraoperativem Befund auch die Gegenseite mitversorgt wird. Alle Patienten wurden 6 Wochen postoperativ klinisch und bei Bedarf auch sonographisch nachkontrolliert. Sämtliche Daten wurden pseudonymisiert in einer klinikinternen Datenbank erfasst, die passwortgeschützt den Untersuchern zugänglich ist. Einseitige Operationen wurden entsprechend dem Schweizer Bundesamt für Gesundheit ambulant-vor-stationär durchgeführt (AVOS). Beidseitige Leistenhernienversorgungen, Rezidiveingriffe und Operationen bei Patienten mit erhöhtem Risikoprofil (z. B. orale Antikoagulation, Gerinnungsstörungen oder nach vorangegangener Prostatektomie) wurden stationär behandelt.

## Ergebnisse

Es wurden 302 Hernien bei 225 Patienten operiert. 87,6 % der Patienten waren männlich. Das Durchschnittliche Alter betrug 58,7 Jahre. Die demographischen Merkmale, Nebenerkrankungen und Risikofaktoren sind in Tab. 1 in drei Zeitabschnitten aufgeführt.

**Table 1:** 

	Total	Erstes Drittel	Zweites Drittel	Drittes Drittel
		05/2018 bis 10/2018	11/2018 bis 04/2019	05/2019 bis 10/2019
	*n* = 225	*n* = 60	*n* = 87	*n* = 78
*Geschlecht*				
Frauen	28 (12,4 %)	4 (6,7 %)	9 (10,3 %)	15 (19,2 %)
Männer	197 (87,6 %)	56 (93,3 %)	78 (89,7 %)	63 (80,8 %)
*Alter in Jahren, MW (Range)*	58,7 (19–95)	58,0 (19–85)	59,4 (24–85)	58,6 (23–95)
*BMI (kg/m*^*2*^*), MW (Range)*	25,5 (16,3–42,6)	25,6 (17,9–34,6)	25,4 (16,3–34,3)	25,5 (17,0–42,6)
*Ethnizität*				
Zentraleuropa	169 (75,1 %)	44 (73,3 %)	67 (77,0 %)	58 (74,4 %)
Mediterran	56 (24,9 %)	16 (26,7 %)	20 (23,0 %)	20 (25,6 %)
*ASA-Klassifikation*				
1	51 (22,7 %)	12 (20,0 %)	16 (18,4 %)	23 (29,5 %)
2	144 (64,0 %)	35 (58,3 %)	63 (72,4 %)	46 (59,0 %)
3	20 (8,9 %)	5 (8,3 %)	7 (8,0 %)	8 (10,3 %)
Unbekannt	10 (4,4 %)	8 (13,3 %)	1 (1,1 %)	1 (1,3 %)
*Komorbiditäten*				
Arterielle Hypertonie	87 (38,7 %)	23 (38,3 %)	37 (42,5 %)	27 (34,6 %)
Diabetes mellitus	19 (5,8 %)	7 (11,7 %)	7 (8,0 %)	5 (6,4 %)
COPD	7 (3,1 %)	2 (3,3 %)	3 (3,4 %)	2 (2,6 %)
Koronare Herzkrankheit	16 (7,1 %)	5 (8,3 %)	4 (4,6 %)	7 (9,0 %)
Nikotinabusus	67 (29,8 %)	22 (36,7 %)	26 (29,9 %)	19 (24,4 %)
Orale Antikoagul	8 (3,6 %)	2 (3,3 %)	3 (3,4 %)	3 (3,8 %)
ASS/Clopidogrel	30 (13,3 %)	11 (18,3 %)	12 (13,8 %)	7 (9,0 %)

*ASA* American Society of Anesthesiologists, *ASS* Acetylsalicylsäure, *BMI* Body-Mass-Index, *COPD* „chronic obstructive pulmonary disease“, *MW* Mittelwert

Die Mehrheit der operierten Hernien waren primäre Hernien, wobei der Anteil an operierten Rezidivhernien im dritten Drittel des untersuchten Zeitraums auf 21,8 % anstieg (Tab. 2). Bei jedem 4. Patienten zeigte sich auf der operierten Seite zusätzlich zur Leistenhernie ein weiterer Befund (Femoral‑, Obturator- oder Spieghel-Hernie). Bei den inguinalen Hernien fand sich am häufigsten eine laterale (L2-)Hernie, gefolgt von L1- und M2-Hernien. Die Mehrzahl der Hernien wurde mit einem 10 × 15 cm großen Netz versorgt (90,4 %). Die größeren Netze (12 × 17 cm) wurden über die 18 Monate betrachtet mit zunehmender Häufigkeit verwendet (2,5 % im 1. Drittel, 11,2 % im 3. Drittel). Die Netze wurden zu Beginn noch selten fixiert; mit zunehmender Erfahrung sowie unter voller Ausnützung der feinmotorischen Möglichkeiten der Robotik wurde die Netzfixation mit resorbierbarer Naht mit Luftknoten an 4 Punkten in die „standard operating procedure“ (intraoperative Checkliste) übernommen (Tab. 2). Die Operationszeit vom Schnitt bis zur Hautnaht (inkl. Adhäsiolyse in einzelnen Fällen) betrug für einseitige Hernien im Durchschnitt 71 min (Range 40–186), für beidseitige Hernien 103 min (Range 58–193) und für einseitige Rezidivhernien 95 min (Range 54–186). Die Zeit vom Beginn des Pneumoperitoneums (Schnitt) bis zum Beginn der Arbeit an der Konsole betrug im Durchschnitt 7 min (Range 4–12). Die einseitige Raffung der Fascia transversalis nahm im Durchschnitt 06:20 min in Anspruch (Range 02:49–10:15). Die einseitige Fibrinkleberversiegelung des Leistenkanals dauerte im Durchschnitt 03:47 min (Range 02:17–04:53). Die Netzfixation mit 4 resorbierbaren Nähten nahm im Durchschnitt 05:17 min in Anspruch (Range 02:05–09:35).

**Table 2:** 

	Total	Erstes Drittel	Zweites Drittel	Drittes Drittel
		05/2018–10/2018	11/2018–04/2019	05/2019–10/2019
	Patienten *n* = 225	*n* = 60	*n* = 87	*n* = 78
*Hernienseiten*	302 (100 %)	80 (100 %)	115 (100 %)	107 (100 %)
Patienten einseitig	148 (65,8 %)	40 (66,7 %)	59 (67,8 %)	49 (62,8 %)
Patienten beidseitig	77 (34,2 %)	20 (33,3 %)	28 (32,2 %)	29 (37,2 %)
*Primäre Leistenhernien*	269 (89,1 %)	75 (93,8 %)	104 (90,4 %)	90 (84,2 %)
*Rezidivleistenhernien*	33 (10,9 %)	5 (6,2 %)	11 (9,5 %)	17 (15,8 %)
*Operationszeit in Minuten*^*a*^*, MW (Range)*				
Einseitige Operation	70,8 (40–186)	66,6 (40–94)	71,6 (41–131)	73,3 (45–186)
Beidseitige Operation	103,9 (58–193)	97,8 (68–159)	112,3 (75–193)	99,6 (58–140)
Rezidivleistenhernie	95,5 (54–186)	90 (54–139)	98,9 (54–169)	94,9 (60–186)
Nach Prostatektomie	86	86	–	–
*Lehreingriffe (n)*	110 (48,9 %)	12 (20,0 %)	51 (58,6 %)	47 (60,3 %)
*Anzahl Befunde pro Seite*				
0	1 (0,3 %)	0	1 (0,9 %)	0
1 (einfach)	240 (79,5 %)	64 (80,0 %)	94 (81,7 %)	82 (76,6 %)
2 (kombiniert)	47 (15,6 %)	12 (15,0 %)	17 (14,8 %)	18 (16,8 %)
3 (kombiniert)	14 (4,6 %)	4 (5,0 %)	3 (2,6 %)	7 (6,5 %)
Nebenbefund Obturator/Spieghel	8 (2,6 %)	1 (1,3 %)	3 (2,6 %)	4 (3,7 %)
*EHS-Klassifikation*				
M1 + M2	85	26	27	32
M3	11	1	3	7
L1 + L2	212	57	80	75
L3	22	2	12	8
F1 + F2	43	12	13	18
F3	3	1	2	0
*Raffung der Fascia transversalis*^*b*^	55/96 (57,2 %)	13/27 (48,14 %)	18/30 (60,0 %)	24/39 (61,5 %)
*Versiegelung des Leistenkanals*^*c*^	15/324 (6,4 %)	0	0	15/83 (18,0 %)
*Netzgröße*				
10 × 15 cm	278 (92,5 %)	78 (97,5 %)	105 (91,3 %)	95 (88,8 %)
12 × 17 cm	24 (7,9 %)	2 (2,5 %)	10 (8,7 %)	12 (11,2 %)
*Netzfixation*^*d*^				
Keine	87 (28,8 %)	70 (87,5 %)	15 (13,0 %)	2 (1,9 %)
Resorbierbare Naht	215 (92,0 %)	11 (13,5 %)	100 (86,9 %)	105 (98,1 %)
*Dauer des Spitalaufenthalts*				
Ambulant	80 (35,6 %)	14 (23,3 %)	40 (46,0 %)	26 (33,3 %)
1 Nacht	99 (44,0 %)	36 (60,0 %)	29 (33,3 %)	34 (43,6 %)
2 Nächte	37 (16,4 %)	7 (11,7 %)	15 (17,2 %)	15 (19,2 %)
≥ 3 Nächte	9 (4,0 %)	3 (5,0 %)	3 (3,4 %)	3 (3,8 %)

*EHS *European Hernia Society, *MW* Mittelwert

^a^Zeitmessung von Beginn der Anlage des Pneumoperitoneums, über Zielausrichtung des DaVinci Xi, Andocken und Operation (inkl. Raffung der Fascia transversalis bzw. Fibrinkleberversiegelung des Leistenkanals sowie Netzfixation) bis Ende der Hautnaht. Die Zeit vom Beginn des Pneumoperitoneums (Schnitt) bis zum Beginn der Arbeit an der Konsole dauert im Durchschnitt 7 min (Range 4–12)

^b^Die einseitige Raffung der Fascia transversalis dauert im Durchschnitt 06:20 min (Range 2:49–10:15)

^c^Die einseitige Fibrinkleberversiegelung des Leistenkanals dauert im Durchschnitt 03:47 min (Range 02:17–04:53)

^d^Die Netzfixation mit 4 resorbierbaren Nähten dauert im Durchschnitt 05:17 min (Range 02:05–09:35)

Insgesamt 48 % aller Eingriffe waren Lehreingriffe, bei denen ChirurgInnen in Weiterbildung im Sinne „professioneller Arbeitseinheiten“ Teile des Eingriffes selbständig durchgeführt haben [20]. Die Zeiten der Lehreingriffe sind ungekürzt in den dargestellten Operationszeiten abgebildet (Tab. 2). Der Instrumentenverbrauch war auf alle Eingriffe bezogen sehr konstant, in 98 % der Fälle wurden nur die drei geplanten Instrumente monopolare Schere (Hot Shears MCS), Fasszange (Prograsp Forceps) und Nadelhalter (Mega SutureCut Needle Driver) verwendet.

Insgesamt haben 14 Patienten (6,2 %) eine Nachkontrolle abgelehnt oder sind nicht erschienen. Die durchschnittliche Zeit bis zur ersten Nachkontrolle betrug 41,4 Tage (Range 1–168 Tage). Die Mehrheit der Patienten (84,4 %) benötigte nur eine Nachkontrolle (Tab. 3). Postoperative Komplikationen werden in Tab. 3 zusammengefasst. Katheterisierungsbedürftiger Harnverhalt wurde in 8 Fällen beobachtet. Serome wurden in 6,6 % der Fälle beschrieben und mehrheitlich konservativ behandelt; die Serominzidenz betrug im ersten Zeitraum 11,2 % und nahm im dritten Untersuchungszeitraum auf 3,0 % ab (Tab. 3). In 7 Fällen wurde eine Seromaspiration im Ambulatorium vorgenommen. Ein Patient benötigte 6 Monate nach der Indexoperation wegen persistierender Beschwerden eine transinguinale Seromkapselresektion und war anschließend beschwerdefrei; bei diesem Patienten war die Fibrinkleberversiegelung des Leistenkanals nicht durchgeführt worden. Ein postoperatives Hämatom wurde in 10 Fällen (3,3 %) beobachtet, 4 dieser 10 Patienten nahmen Acetylsalicylsäure (ASS) oder Clopidogrel (40 % der Hämatomfälle), während unter den Patienten ohne Hämatom nur 7,8 % ASS oder Clopidogrel einnahmen; bei 2 Patienten wurde eine Hämatomrevision mit Drainageneinlage in Allgemeinnarkose durchgeführt (0,6 %). Es traten keine Wundkomplikationen und kein postoperativer Ileus auf. Bei keinem der Patienten kam es zu Schmerzsymptomen durch Nervenläsion (keine neuropathischen Schmerzen). Bis zum aktuellen Zeitpunkt ist kein Rezidiv aufgetreten.

**Table 3:** 

	Total	Erstes Drittel	Zweites Drittel	Drittes Drittel
		05/2018–10/2018	11/2018–04/2019	05/2019–10/2019
Patienten (Seiten)	*n* = 225 (302)	*n* = 60 (80)	*n* = 87 (115)	*n* = 78 (107)
*Follow-up 6 Wochen*	211 (93,7 %)	55 (91,6 %)	80 (91,9 %)	76 (97,4 %)
Ungeplante WV	9 (4,0 %)	4 (6,7 %)	3 (3,4 %)	2 (2,6 %)
*Komplikationen n (%)*				
*Harnverhalt* (CD 2)	8 (3,6 %)	–	4 (4,6 %)	1
Symptomatisches Serom*	20 (6,6 %)	9 (11,2 %)	7 (6,0 %)	4 (3,6 %)
Konservativ (CD 1)	12 (60,0 %)	6 (66,7 %)	5 (71,4 %)	1 (25,0 %)
Abpunktion (CD 3a)	7 (35,0 %)	2 (22,2 %)	2 (28,6 %)	3 (75,0 %)
Operation (CD 3b)	1 (0,3 %)	1 (1,2 %)	–	–
*Hämatom*	10 (3,3 %)	5 (6,2 %)	3 (2,6 %)	2 (1,8 %)
Konservativ (CD 1)	8 (80,0 %)	5	2	1
Operation (CD 3b)	2 (20,0 %)	–	1	1
*Lungenembolie* (CD 4)	1 (0,3 %)	–	–	1
*TBV* (CD 2)	1 (0,3 %)	–	1	–
*Epididymitis*	7 (2,3 %)	4 (5,0 %)	3 (2,6 %)	–
Antibiotika (CD 2)	7	4	3	–
*Wundheilungsstörung*	**–**	**–**	**–**	**–**
*Postoperativer Ileus*	**–**	**–**	**–**	**–**
*Rezidiv*	**–**	**–**	**–**	**–**

* Es besteht ein signifikanter Trend zu weniger Seromen von der ersten zur letzten Untersuchungsperiode (Chi-Quadrat Test für Trend, p = 0,043).

*CD* Clavien-Dindo-Klassifikation der Komplikationen, *rTAPP* robotische transabdominelle präperitoneale Patchplastik, *TBV* tiefe Beinvenenthrombose, *WV* Wiedervorstellung

## Diskussion

Die für die endoskopische TAPP relevanten Vorteile des robotischen Systems sind folgende: Arbeiten in vergrößertem intraabdominellem Raum auch mit geringem Pneumoperitoneumdruck, standardisierte Arbeitsdistanz zum Zielorgan, Immersionsblick und stabile Kameraführung, Bedienung der Präzisionsinstrumente mit 3:1-Bewegungsübertragung (was bedeutet, dass die Choreographie der Bewegung grobmotorisch, die Ausführung allerdings feinmotorisch erfolgt) und nicht zuletzt der besondere Wert in der Weiterbildung mit Anwendung der doppelten Steuerkonsole. Das Fehlen der Haptik wird durch die positive Bilanz aller o. g. Vorteile weit kompensiert und ist im Alltag kein Thema. Die Expertise, die mit der Bedienung des DaVinci Xi bei der Versorgung von Leistenhernien gewonnen wird, bewährt sich nicht zuletzt bei der Durchführung größerer viszeralchirurgischer Eingriffe. In unserer Klinik gehören dazu neben der tiefen anterioren Rektumresektion mit totaler mesorektaler Exzision (TME) z. B. auch die onkologische Hemikolektomie rechts mit kompletter mesokolischer Exzision (CME), die Gastrektomie mit D2-Lymphadenektomie oder Magenvollwandresektionen bei gastrointestinalen Stromatumoren (GIST) und bariatrische Eingriffe, um nur einige zu nennen.

Vergleiche von robotischen und laparoskopischen Operationstechniken fielen bisher bezüglich operativem Outcome zumeist gleichwertig aus, wobei verminderte postoperative Schmerzen nach robotischen Operationen beschrieben wurden [21, 22]. Im Vergleich zur offenen Leistenhernienoperationen zeigte sich bei robotisch assistierten Operationen eine signifikant geringere Zahl postoperativer Komplikationen und Reoperationen [23]. Das postoperative Serom ist ein noch nicht gelöstes Problem, zu dessen Prophylaxe verschiedene Strategien evaluiert wurden [24]. Auf die morphologische Wiederherstellung der Hinterwand des Leistenkanals wurde bei konventionellen endoskopischen Verfahren historisch aus technischen Gründen immer wieder verzichtet, dies ist jedoch robotisch keine besondere Herausforderung mehr. Die Nahtraffung der Fascia transversalis wurde in unserem Kollektiv immer häufiger durchgeführt und wird aktuell nur bei sehr kleinen Befunden nicht gemacht. Pini et al. haben an 61 r‑TAPP-Seiten die Fascia transversalis mit V‑Loc-Naht gerafft und nach 30 Tagen weder ein Serom noch ein Rezidiv sowie keine prolongierten oder chronischen Schmerzen in einem Follow-up von 10 Monaten beobachtet [25]. Zur Fibrinkleberversiegelung liegt eine randomisierte Studie vor, die an 40 Patienten zeigt, dass die Seromprophylaxe signifikant ist (*p* < 0,001; [26]). In der aktuellen Serie sind 17 von 20 Seromen bei lateralen Hernien beobachtet worden, von denen nur 1 Patient mit Fibrinkleber versiegelt worden war. Weitere randomisierte Studien sind nötig, um die positive Korrelation der Fibrinkleberversiegelung mit der Reduktion der Serominzidenz zu belegen oder abzuweisen.

Lernkurven versuchen zu erfassen, nach wie vielen Operationen ein Indikator, z. B. die Operationszeit, ein stabiles Plateau erreicht. Im Kontext der Robotik im Weiterbildungsspital sind immer zwei Lernkurven gleichzeitig zu bewältigen: die prozedurspezifische Lernkurve (die TAPP-Operation an und für sich) und die Beherrschung des Roboters. Die Lernkurve der r‑TAPP ist steil und die beschriebenen Reduktionen in der Operationszeit zeugen von der raschen und intuitiven Annahme der Vorteile der Robotik [27, 28]. In einer Studie zur rTAPP aus Italien wurde berechnet, dass nach 43 r‑TAPPs durch erfahrene ChirurgInnen die Lernkurve das Plateau erreicht (von 70 min auf 61 min; [29]). Die r‑TAPP-Lernkurve des europäischen Pioniers der robotischen Hernienreparation Filip Muysoms aus Belgien zeigt ähnliche Ergebnisse: In einem Jahr wurde die Operationszeit (Schnitt-Naht) von 80 min auf 60 min gesenkt; bei Muysoms et al. beträgt die durchschnittliche Konsolenzeit für einseitige Hernien 43 min (gesamte Operationszeit 94 min), für beidseitige Hernien 65 min (gesamte Operationszeit 119 min; [27]).

Aus unseren Daten ist neu die stabile Operationszeit bei Weiterbildungseingriffen mit der doppelten Konsole ersichtlich (Abb. 5). Diese Lernkurve mag drei Gründe haben: a) Wir haben uns von Anfang des Robotikprogramms an entschieden, die prozedurspezifische Lernkurve am Patienten zu minimieren, b) die Roboter-Lernkurve am Simulator zu absolvieren, indem jeder Operateur vor Beginn an der Konsole mindestens 20 h am Simulator üben musste, und c) durch Arbeitsteilung im Sinne „professioneller Arbeitseinheiten“ kann es sein, dass junge ChirurgInnen unter Supervision und abwechselnden Konsolenübergaben an mehreren Patienten hintereinander den Zugang machen, dann mehrmals hintereinander die Bruchpforten bearbeiten und wiederum mehrere Male das Netz positionieren, fixieren und das Peritoneum vernähen. Wir verfügen am KSO über einen eigenen Simulator, an dem das Team kontinuierlich trainiert wird. Ein möglicher Grund, warum die Operationszeit erfahrener Operateure nicht weiter reduziert wird, ist, dass ChirurgInnen mehr Zeit im Dialog mit dem Gewebe verbringen, da mehr Einzelheiten wahrgenommen werden und ergonomische Rücksicht nicht nötig ist. Ein zweiter Grund ist, dass immer komplexere Fälle mit dem DaVinci Xi operiert werden, was sich auch an den Zahlen stationärer Patienten merkbar macht (Tab. 2). Die Zeit zwischen zwei Operationen variiert je nach Komorbiditäten von 18–35 min.

**Figure 6:**
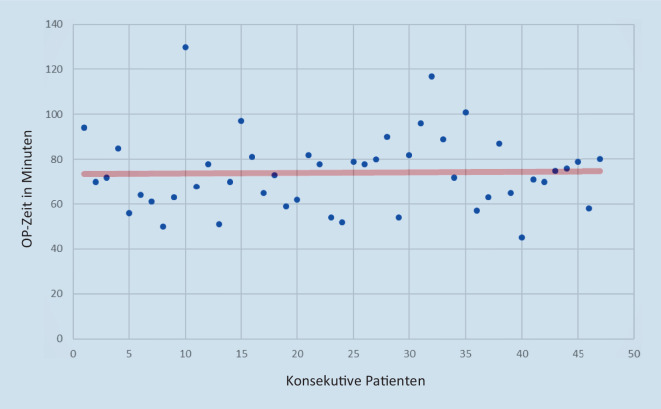

Eine Limitation der aktuellen Studie ist das bisherige Fehlen der Langzeitergebnisse zur Inzidenz des Rezidivs; auch eine genauere Interpretation der prolongierten (nichtneuropathischen) postoperativen Schmerzen, vor allem unter nichtnordeuropäischen Patienten, steht noch aus. Eine weitere Limitation ist, dass wir zwar bei zunehmender Expertise und Anpassung der Operationstechnik weniger Serome beobachtet haben, ob jedoch die Raffung der Fascia transversalis und die Fibrinkleberversiegelung einen positiven Effekt auf die Seromreduktion haben, muss in einer zukünftigen prospektiven randomisierten Studie geklärt werden.

In der Schweiz entstehen mit der r‑TAPP Kosten von 950 CHF für das Material und 420 CHF Umlage der Wartungspauschale (bei 300 Eingriffen/Jahr) je Patienten (die Kosten sind auch für Deutschland bei einem Wechselkurs von ca. 1:1 übertragbar). Bei zum Beispiel 10.000 Eingriffen im Jahr und 8 Mio. gesetzlich versicherten Einwohnern würde die robotische Versorgung sämtlicher Leistenhernien die Allgemeinheit mit einem Zusatzbetrag von 14 Rappen/Monat oder 1,71 CHF/Jahr bzw. einer halben Tasse Espresso/Jahr/Versicherten belasten. Zukünftige Studien sind notwendig, um zu zeigen, ob die durch die Robotik erwartete Verbesserung der Ergebnisse reproduzierbar ist; wenn ja, würden diese nicht nur die Kosten kompensieren, sondern vor allem die Lebensqualität des einzelnen Patienten positiv beeinflussen.

Zu argumentieren, die r‑TAPP sei unnötig, weil sie auf einen ersten Blick keine Vorteile habe und zu teuer sei, zeugt entweder von Unkenntnis der Methode oder Leugnung der historischen Durchsetzungskraft technologischer Fortschritte gerade auch in der Viszeralchirurgie [30]. Die Chirurgie der Leistenhernien wird mit an Sicherheit grenzender Wahrscheinlichkeit nie zu einem abschließenden Kapitel kommen. Zum einen, weil die Reparationsintervention auch in Zukunft kaum auf Genomebene erfolgen, sondern immer eine anatomisch-chirurgische bleiben wird, der Fortschritt unaufhaltsam weitergeht und präzisere Instrumente immer wieder neu den Umgang mit dem Gewebe revolutionieren werden; zum anderen aber auch weil die Heterogenität der Hernienbefunde dem vorzeitigen Abschluss dieses Kapitels noch über Generationen trotzen wird.

## Fazit für die Praxis

- Erweiterte anatomische Kenntnisse des myopektinealen Trichters sind für die robotische transabdominelle präperitoneale Patchplastik (r‑TAPP) unabdingbar.
- Raffung der Fascia transversalis, Fibrinkleberversiegelung des Leistenkanals und Nahtfixation des Netzes sind zusätzliche Arbeitsschritte, deren Mehrwert in zukünftigen Studien gezeigt werden muss.
- Das Arbeiten mit doppelter Operationskonsole bietet optimale Bedingungen für die Weiterbildung, ohne prozedurbedingte Lernkurve am Patienten und mit planbaren Operationszeiten.
- Die postoperative Serombildung und die Komplikationsrate der r‑TAPP sind gering.
- Die r‑TAPP ist die natürliche Weiterentwicklung der konventionellen TAPP, ihre Akzeptanz wird proportional zur Geräteverfügbarkeit und Kostensenkung wachsen.

## Supplementary Information




