# Erratum zu: Robotische Hernienchirurgie I: Robotische Leistenhernienversorgung (r-TAPP). Videobeitrag und Ergebnisse einer Kohortenstudie an 302 operierten Hernien

**DOI:** 10.1007/s00104-022-01571-5

**Published:** 2022-01-24

**Authors:** Michaela Ramser, Johannes Baur, Nicola Keller, Jan F. Kukleta, Jörg Dörfer, Armin Wiegering, Lukas Eisner, Ulrich A. Dietz

**Affiliations:** 1 Klinik für Viszeral‑, Gefäss- und Thoraxchirurgie, Kantonsspital Olten, Baslerstr. 150, 4600 Olten, Schweiz; 2 Klinik für Allgemein‑, Viszeral- und Gefässchirurgie, Kantonsspital Baden, Im Engel 1, 5404 Baden, Schweiz; 3 Hernienzentrum Zürich, Grossmünsterplatz 9, 8001 Zürich, Schweiz; 4 Klinik und Poliklinik für Allgemein‑, Viszeral‑, Transplantations‑, Gefäß- und Kinderchirurgie, Universitätsklinikum Würzburg, Oberdürrbacher Str. 6, 97080 Würzburg, Deutschland

**Erratum zu:**

**Chirurg 2021**

10.1007/s00104-021-01425-6

Dieser Beitrag zum Thema „Robotische Hernienchirurgie. R‑TAPP“ ist der erste Teil einer Reihe von mehreren Beiträgen zum Thema. Titel und Untertitel wurden angepasst. Die Originalversion dieses Beitrags wurde korrigiert. 

